# Transient transformation meets gene function discovery: the strawberry fruit case

**DOI:** 10.3389/fpls.2015.00444

**Published:** 2015-06-12

**Authors:** Michela Guidarelli, Elena Baraldi

**Affiliations:** Laboratory of Plant Pathology and Biotechnology, Department of Agricultural Sciences, University of BolognaBologna, Italy

**Keywords:** strawberry, *Agrobacterium*-mediated transient transformation, gene function discovery, RNA-interference, overexpression

## Abstract

Beside the well known nutritional and health benefits, strawberry (*Fragaria* X *ananassa*) crop draws increasing attention as plant model system for the *Rosaceae* family, due to the short generation time, the rapid *in vitro* regeneration, and to the availability of the genome sequence of *F.* X *ananassa* and *F. vesca* species. In the last years, the use of high-throughput sequence technologies provided large amounts of molecular information on the genes possibly related to several biological processes of this crop. Nevertheless, the function of most genes or gene products is still poorly understood and needs investigation. Transient transformation technology provides a powerful tool to study gene function *in vivo*, avoiding difficult drawbacks that typically affect the stable transformation protocols, such as transformation efficiency, transformants selection, and regeneration. In this review we provide an overview of the use of transient expression in the investigation of the function of genes important for strawberry fruit development, defense and nutritional properties. The technical aspects related to an efficient use of this technique are described, and the possible impact and application in strawberry crop improvement are discussed.

## Genetic Analysis and Gene Function Discovery

Strawberry is an important fruit crop worldwide, both for the impact on the economy of several countries and for its nutritional properties highly beneficial to human health. Strawberry is considered a plant model system for *Rosaceae* family due to its small genome size, the short generation time for a perennial species (Folta and Davis, 2006), the easy *in vitro* regeneration and transformation system (Barceló et al., 1998; Folta et al., 2006; Debnath and Teixeira da Silva, 2007; Qin et al., 2008) and the current availability of different genome sequences of *Fragaria* species. These include the diploid *F. vesca* genome (Shulaev et al., 2011) and a virtual strawberry reference genome, named ‘FANhybrid_r1.2,’ obtained by integrating the four homoeologous subgenomes sequences of the octoploid *F.* X *ananassa* (Hirakawa et al., 2014). In the past, different transcriptomic approaches have been used to highlight genes regulated during fruit development or upon pathogen interaction. These included microarray experiments, cDNA libraries sequencing, and suppressive subtractive hybridization (SSH) technique. These provided a large set of expressed sequence tags (ESTs) from *F. vesca* and *F.* X *ananassa* related to candidate genes involved in important biological processes for strawberry (Medina-Escobar et al., 1997; Aharoni and O’Connell, 2002; Aharoni et al., 2002; Salentijn et al., 2003; Casado-Dìaz et al., 2006; Bombarely et al., 2010; Folta et al., 2010; Guidarelli et al., 2011). More recently, the new RNA sequencing technologies offered the opportunity to uncover whole transcriptome changes underneath complex biological processes as, for example, the strawberry flower and fruit development (Kang et al., 2013; Hollender et al., 2014). The large amount of gene expression data derived by these techniques is generally thoroughly processed with the available bioinformatics tools that allow visualizing all the transcriptome networks controlling a specific function in comprehensive pictures. These are mostly based on the predictive gene functional classification derived by gene homology scores, and only for a little percentage on the experimental evidence of a specific gene function. Indeed, to fully understand the role of each gene involved in a biological process an *in vivo* genetic analysis should be performed. Before the advent of the whole genome sequencing, gene function analysis was carried out using ‘forward’ genetics, where the genetic basis of a phenotype was identified by associating the phenotypic effects of natural or induced mutations to a specific gene sequence. At present, thanks to the new sequencing technologies and the consequent massive availability of gene sequences, it is possible to explore gene function in the opposite direction, namely from gene to phenotype, through the so-called ‘reverse’ genetics. Here, a specific gene with a known sequence is disrupted or modified and the phenotype is analyzed to determine the corresponding gene function (Tierney and Lamour, 2005). For plants several approaches have been developed for this purpose, including overexpression, gene silencing or mutagenesis (Gilchrist and Haughn, 2010).

In particular for strawberry, reverse genetics has been successfully used to characterize gene function mainly through gene down-regulation, via post-transcriptional gene silencing (PTGS) RNA interference (RNAi). This can be achieved through the introduction of a double-stranded RNA (dsRNA) homologous in sequence to the gene of interest, resulting in the degradation of the target gene transcripts and leading to a knockout or a knockdown phenotype, depending on the specific silencing efficiency (Filipowicz et al., 2005). The silence-inducing dsRNA can be delivered in host plant cells either by the introduction of plasmid constructs encoding self-complementary ‘hairpin’ RNAs, or by Virus-induced gene silencing (VIGS). The latter is obtained by cloning a cDNA fragment of the gene of interest into a DNA copy of a RNA-virus genome, resulting in the production of an autonomous replicating virus, which, during its replication in plant, forms a dsRNA (Lu et al., 2003). However, most viruses used for VIGS have a limited number of hosts and the efficiency of silencing is strongly dependent on the virus-host affinity. Furthermore, the phenotypes introduced with this technique are not heritable and cannot be used for obtaining stable genetic lines (Gilchrist and Haughn, 2010). On the contrary, the phenotype introduced by hairpin RNAi-based silencing can be heritable, moreover the transcripts of multigene families can be silenced by a single construct (Alvarez et al., 2006), making this technique a powerful tool to study the loss of function phenotype of a target gene in plants with high levels of polyploidy such as strawberry.

Similarly, gain of function phenotypes, which are achievable simply by inducing the expression of a specific gene under the control of a strong promoter, have also been used to efficiently study gene function in strawberry (Agius et al., 2005; Hoffmann et al., 2011; Cumplido-Laso et al., 2012). On the other hand, the utility of the overexpression system is limited because the ectopic protein expression ceases after few days due to PTGS. In *Nicotiana benthamiana*, the viral protein P19 was shown to prevent the onset of PTGS allowing high level of transient expression of genes (Voinnet et al., 2003). Aragüez et al. (2013) used P19 to coexpress *FaEGS2*, a gene involved in the formation of eugenol and isoeugenol, and to evaluate its effect in the aroma of strawberry. In strawberry, the use of this factor could be very useful to reduce PTGS, however, is still very limited; more studies are needed to deeply evaluate the co-expression of P19 together with target gene over-expression, thus confirming its role as gene expression enhancer, and ultimately allowing overcoming the problem of the PTGS in this type of experiments.

Nevertheless, RNAi and overexpression systems, represent the most powerful tools to investigate gene function in plants and consequently to improve beneficial traits such as novel disease resistances, quality and nutritional improvements, and changes in metabolism which will increase crop productivity.

## Advantages of Transient Transformation

Reverse genetics can be accomplished through stable transformation or transient expression of a target gene. However, generation of stably transformed plants is difficult and labor-intensive, and represents a relatively low-throughput process lasting several months, depending on the plant species used. In comparison with stable transformation, the transient transformation has several advantages: (i) it does not interfere with the stability of the host genome (Lu et al., 2013), (ii) it does not require regeneration of a transformed cell in order to analyze the transformation, (iii) the expression of target genes can be analyzed shortly after DNA delivery, and (iv) is not influenced by positional effects. Moreover, transient transformation systems can greatly accelerate research timing, since many constructs can be analyzed in parallel within a short time frame (Li et al., 2009). These advantages make this system a powerful method especially in plants that are recalcitrant to regeneration (Kapila et al., 1997) and can be widely exploited for studying gene functions through physiological characterization of the phenotype and cellular localization of gene product (Koroleva et al., 2005; Kirienko et al., 2012). Routine transient assays include biolistic bombardment (Christou, 1995), protoplast tranfection (Sheen, 2001), and *Agrobacterium*-mediated transient assays (Kapila et al., 1997; Yang et al., 2000) each with advantages and disadvantages depending on the research goals: the biolistic approach can be used on various plant species but it requires an expensive particle bombardment equipment and a procedure relatively complex, in particular for the case of ripe fleshy fruits, anatomically not so suitable to bombardment. Similarly, protoplast transfection works well for several plant species and could be useful to investigate cell-autonomous regulatory processes and responses in a quantitative and high-throughput way (Yoo et al., 2007). However, protoplast assay has limited applicability for those cases where the cell wall or a tissue context is required (Li et al., 2009). On the contrary, *Agrobacterium*-mediated transformation represents one of the most facile and effective methods to transfer a gene in plant cells and to analyze its function (Gelvin, 2003). Here, the common gall-inducing bacterium *Agrobacterium tumefaciens* is used to transfer a DNA segment (T-DNA) from the bacterial tumour-inducing (Ti) plasmid to plant cell. The target gene (either for overexpression or for RNAi silencing), inserted into the T-DNA depleted of tumor-inducing genes, can be efficiently expressed under the control of a eukaryotic promoter. Once moved into the plant cell, the T-DNA migrates to the nucleus and only a tiny part is integrated into the host chromosomes; these not-integrated T-DNA copies persist in the nuclei of transfected cells remaining transcriptionally competent for several days and leading to non-stable, transient transcripition (Hellens et al., 2005). This technique, commonly known as agroinfiltration or agroinjection, provides fast and efficient ways to transiently express or silence a desired gene whose function must be investigated ‘*in planta.*’

## The Strawberry Fruit Case

In the past, functional genomics in strawberry was mainly confined to gene expression analysis and *in silico* prediction. Only recently, reverse genetics approaches were used to assign gene functions (Schwab et al., 2011). A number of genetically transformed lines of *Fragaria* sp. were obtained throughout genetic engineering to study gene functions or to improve traits such as resistance, ripening, and fruit production (Haymes and Davis, 1998; Mezzetti et al., 2004; Schaart et al., 2004, 2013; Oosumi et al., 2006; Jiwan et al., 2013; Pantazis et al., 2013; see also reviews: Folta and Davis, 2006; Aldwinckle and Malnoy, 2009). Indeed, in strawberry the efficiency of transformation and the regeneration capacity is highly dependent on the genetic background (Landi and Mezzetti, 2006) and several critical factors such as hormones concentrations, incubation conditions, explant source, antibiotic, and mode of transfections can affect the frequency of regeneration (Folta and Dhingra, 2006). In addition, these traditional ways of studying gene functions in strawberry are very time-consuming, especially for ripening-related genes, considering that, from the transformation experiment until the first ripe fruits become available for analyses, at least 15 months are needed (Hoffmann et al., 2006). For these reasons, more recently, transient transformation systems are emerging as most powerful tool to investigate the function of genes involved in important physiological processes, such as ripening related genes, resistance genes, and strawberry promoters (**Table 1**).

**Table 1:** Genes functionally characterized through transient transformation in strawberry.

Gene	Transient method	Putative function	Reference
**Ripening**		
Calchone synthase (FaCHS)	RNA interference (RNAi): intron-hp construct	Pigment formation, flavonoid biosynthesis. Calchone synthase (CHS) silencing lead to an increase in lignin content	Hoffmann et al. (2006), Miyawaki et al. (2012), Ring et al. (2013)
Glycosyltransferase (FaGT1)	RNAi: intron-hp construct	Pigment formation, flavonoid biosynthesis	Griesser et al. (2008)
Pathogenesis related Fra a1	RNAi: intron-hp construct	Pigment formation, flavonoid biosynthesis	Muñoz et al. (2010)
Eugenol synthase (FaEGS)	Overexpression	Aroma, phenylpropene synthesis	Hoffmann et al. (2011)
Isoeugenol synthase (FaIGS)	Overexpression	Aroma, phenylpropene synthesis	Hoffmann et al. (2011)
Eugenol synthase (FaEGS1a)	Overexpression	Aroma, phenylpropene synthesis	Aragüez et al. (2013)
Eugenol synthase (FaEGS1b)	Overexpression	Aroma, phenylpropene synthesis	Aragüez et al. (2013)
Eugenol synthase (FaEGS2)	Overexpression	Aroma, phenylpropene synthesis	Aragüez et al. (2013)
Alcohol acyltransferase (FaAAT2)	Overexpression	Aroma, fruit ester formation	Cumplido-Laso et al. (2012)
Epoxycarotenoid dioxygenase (FaNCED1)	RNAi: VIGS	Pigment formation, ABA biosynthesis	Jia et al. (2011)
FaCHLH/ABAR	RNAi: VIGS	ABA receptor gene regulating ABA signaling	Jia et al. (2011)
FaPYR1	RNAi: VIGS	ABA receptor gene regulating ABA signaling	Chai et al. (2011)
Sucrose transporter (FaSUT1)	RNAi: intron-hp construct; overexpression	Regulation of sucrose, ABA content and fruit ripening	Jia et al. (2013)
FaSHP transcription factor	RNAi: intron-hp construct; overexpression	Regulation of ripening time	Daminato et al. (2013)
FaMYB transcription factor	RNAi: intron-hp construct. Intron-hp construct /overexpression	Regulation of phenylpropanoid/flavonoid pathways, eugenol production Pigment formation, flavonoid biosynthesis	Kadomura-Ishikawa et al. (2014), Medina-Puche et al. (2014, 2015)
Flavanone 3-Hydroxylase (F3H)	RNAi: intron-hp construct	Pigment formation, flavonoid biosynthesis	Jiang et al. (2013)
Phototropin (FaPHOT2)	RNAi: intron-hp construct; overexpression	Pigment formation	Kadomura-Ishikawa et al. (2013)
β–glucosidases (FaBG3)	RNAi: VIGS	Modulation of ABA, sugar, ripening related genes	Li et al., 2013
Rhamnogalacturonate lyase (FaRGLyase1)	RNAi: intron-hp construct	Softening, pectin degradation	Molina-Hidalgo et al. (2013)
Dihydroflavonol 4-reductase (FaDFR)	RNAi: intron-hp construct	Pigment formation, flavonoid biosynthesis	Lin et al. (2013)
FaGAST2	RNAi: intron-hp construct	Arresting cell elongation	Moyano-Cañete et al. (2013)
Cinnamoyl-CoA reductase (CCR)	RNAi: intron-hp construct; overexpression	Lignin biosynthesis, firmness	Yeh et al. (2014)
Cinnamyl alcohol dehydrogenase (CAD)	RNAi: intron-hp construct; overexpression	Lignin biosynthesis, firmness	Yeh et al. (2014)
Peroxidase (POD27)	RNAi: intron-hp construct; overexpression	Lignin biosynthesis, firmness	Yeh et al. (2014)
**Resistance**		
β–glucosidases (FaBG3)	RNAi: VIGS	Defense to *Botrytis cinerea*	Li et al. (2013)
Mannose binding lectin (FaMBL1)	RNAi: intron-hp construct; overexpression	Defense to *Colletotrichum acutatum*	Guidarelli et al. (2014)
**Promoters**		
Endo-β-1,4-glucanase (FaEG1)	Overexpression	Biotechnological interest	Spolaore et al. (2003)
Endo-β-1,4-glucanase (FaEG3)	Overexpression	Biotechnological interest	Spolaore et al. (2003)
D-galacturonate reductase (GaIUR)	Biolistic overexpression	Biotechnological interest	Agius et al. (2005)

The first report of transient transformation in strawberry fruits goes back to 2001 when Spolaore et al. (2001), showed that strawberry fruit is suitable to transient gene expression mediated by *Agrobacterium* using the β-glucuronidase (GUS) reporter gene interrupted by an intron. Consistently, this system has been firstly used for the functional analysis of two strawberry β*-1-4 glucanases* promoters or, combined with a biolistic transformation protocol, to functionally characterize other homologous and heterologous promoters (Spolaore et al., 2003; Agius et al., 2005).

A number of transient transformation studies on strawberry fruits were subsequently performed, the majority of which were finalized to understand the role of genes involved in ripening processes (**Table 1**). Strawberry fruit ripening is accompanied by an increase of flavonoid biosynthesis resulting in anthocyanin accumulation at ripe red stage (Almeida et al., 2007). *Chalcone synthase* (*CHS*), the key gene in the flavonoid pathway, was chosen as a reporter gene to set up an efficient protocol for RNAi-based transient gene silencing in strawberry fruit: by agroinjecting receptacles with an inoculum of *Agrobacterium* carrying an intron-containing self-complementary hairpin construct, a drastic reduction in mRNA levels and enzymatic activity of *CHS* gene was obtained, leading to the loss of pigmentation in ripe strawberry fruits (Hoffmann et al., 2006). This transient RNAi method was successfully employed afterward for the silencing of other flavonoids related genes and found to be applicable also to strawberry fruits at postharvest stage (Schwab et al., 2011; Miyawaki et al., 2012).

**FIGURE 1:**
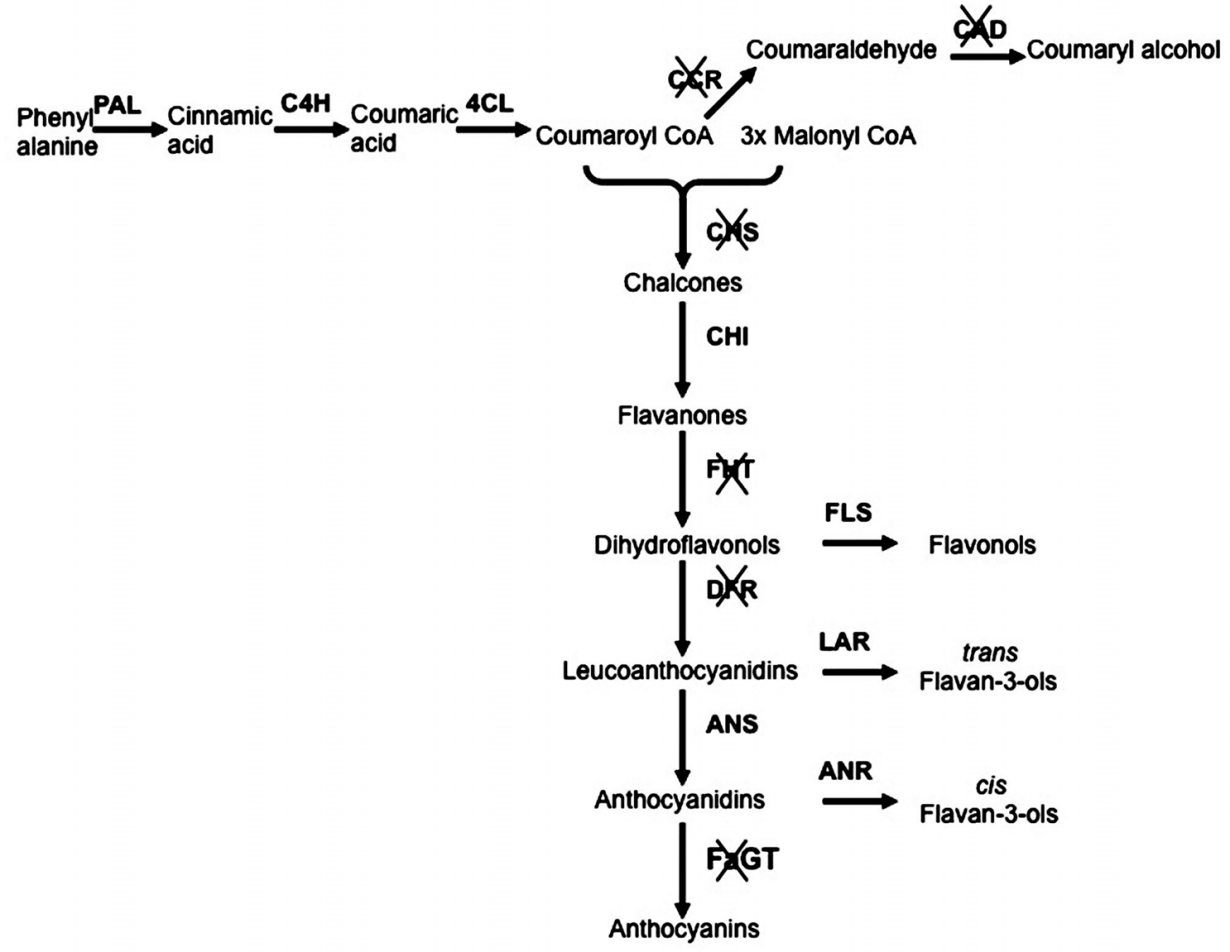
**Schematic representation of phenylpropanoid/flavonoid pathway with silenced genes**. The genes functionally investigated throughout RNA interference (RNAi)-transient transformation are crossed. Genes abbreviations: PAL, phenylalanine ammonia lyase; C4H, cinnamic acid 4-hydroxylase; 4CL, p-coumarate:CoA ligase; CCR, Cinnamoyl-CoA reductase; CAD, Cinnamyl alcohol dehydrogenase; CHS, Calchone synthase; CHI, chalcone isomerase; FHT, Flavanone 3-Hydroxylase; FLS, flavonol synthase; DFR, Dihydroflavonol 4-reductase; LAR, leucoanthocyanidin reductase; ANS, anthocyanidin synthase; ANR, anthocyanidin reductase; FaGT, flavonoid glycosyltransferases.

With the aim to elucidate *in planta* function of *Anthocyanidin 3-0-glycosyltransferase* (*FaGT1)*, a gene encoding for the enzyme involved in the anthocyanin biosynthesis, an *Agrobacterium* carrying a T-DNA expressing an RNAi-*FaGT1* construct was injected into midsized ripening strawberry fruits. This resulted in reduced concentrations of anthocyanin pigments and in a significant increase in epiafzelechin, the compound sinthesized by the anthocyanidin reductase (ANR) enzyme, indicating competition between FaGT1 and FaANR for the common athocyanidin substrate and showing that *FaGT1* is a key gene in the anthocyanin pathway (Griesser et al., 2008). More recently others RNAi studies were performed to clarify the role of other important genes involved in the strawberry fruit pigmentation and anthocyanin biosynthesis (Jiang et al., 2013; Lin et al., 2013; Kadomura-Ishikawa et al., 2014; Medina-Puche et al., 2014). These works greatly helped to clarify the flavonoid pathway and to highlight the contribution of each gene to the synthesis of key compounds, important not only for the agronomic but also for the medical and nutritional interest of this crop (**Figure 1**).

Furthermore, Muñoz et al. (2010) found a correlation between a strawberry pathogenesis-related 10 (PR-10) gene and anthocyanin biosynthesis. In particular, a *Fra a1* gene, was RNAi-silenced, leading to a decreased endogenous concentration of the main flavonoids responsible for the red color of fruits and to a decrease of the expression levels of genes encoding for phenylalanine ammonia lyase (PAL) and CHS. These results demonstrated that *Fra a1* is directly linked to flavonoid biosynthesis and that its function may be regulatory (Muñoz et al., 2010). Consistent to this, very recently, Casañal et al. (2013) demonstrated that strawberry Fra proteins bind flavonoids, providing an important contribution to the function of these PR proteins in the control of flavonoid metabolism.

Gene function in strawberry has been investigated with transient transformation not only through gene silencing but also through gene overexpression of target genes or by combining, alternatively or simultaneously, overexpression with silencing and by observing the consequent phenotype (Hoffmann et al., 2011; Cumplido-Laso et al., 2012; Aragüez et al., 2013; Daminato et al., 2013; Jia et al., 2013; Kadomura-Ishikawa et al., 2013, 2014; Guidarelli et al., 2014; Yeh et al., 2014). For instance, the role of *Eugenol synthase (EGS)* genes in contributing to volatile compounds production responsible for strawberry flavor was studied by modifying the anthocyanin biosynthesis pathway in strawberry fruit using a simultaneous downregulation and ovexpression of *CHS* and *EGS* genes, respectively. This led to the deviation of the flavonoid pathway to the synthesis of phenylpropene, such as eugenol, confirming the role of EGS enzyme in the production of strawberry aroma (Hoffmann et al., 2011; Aragüez et al., 2013). Furthermore, in a recent study, Medina-Puche et al. (2015) used *FaEOBII*-ripe silenced strawberry fruits to prove that eugenol production is regulated by the R2R3-MYB transcription factor *FaEOBII*.

Similarly, by using independent transient overexpression/silencing methods or by combining them, the role of a *FaPOD27* peroxidase gene was found to contribute to lignin biosynthesis and strawberry fruit firmness (Ring et al., 2013; Yeh et al., 2014).

If a large number of experiments were aimed at elucidating the role of genes involved in strawberry ripening, very little with transient transformation has been done for understanding the role of genes involved in strawberry resistance to stress.

By using a VIGS RNAi approach, a β*-glucosidase* gene (*FaBG3*) was found to be involved in the resistance of strawberry to *Botrytis cinerea* (Li et al., 2013); silencing of this gene led to a decrease of endogenous abscisic acid (ABA) and consequent fruit ripening inhibition, but also concomitantly to resistance to the *B. cinerea* fungus and increase in PAL activity and content of phenolic compounds. These results indicated that this gene plays roles not only in fruit growth regulation but also in the fruit response to pathogens. More recently, *Agrobacterium* transient transformation was successfully applied to study the role of the gene *FaMBL1*, encoding for a Mannose Binding Lectin. Expression of this gene was originally found upregulated exclusively in unripe strawberries upon infection with the pathogen *Colletotrichum acutatum*, but not in infected ripe ones. Since, contrary to red ripe fruits, white unripe fruits are reluctant to *C. acutatum* infection, a role for this lectin gene in the different susceptibility of strawberries during ripening was hypothesized. To test this hypothesis, *Agrobaterium*-mediated transient transformation was performed to silence and overexpress *FaMBL1* gene in unripe and ripe strawberries, respectively, before *C. acutatum* infection. *FaMBL1*-silenced unripe fruits showed anthracnose symptoms, whereas *FaMBL1*-overexpressing ripe strawberries presented a lower susceptibility to *C. acutatum*. These experiments allowed concluding that this gene plays a crucial role in the resistance of unripe strawberry fruits to *C. acutatum* (Guidarelli et al., 2014).

Particular attention musts be paid while using *Agrobacterium*-mediated transient transformation for studying plant pathogen interactions: *Agrobacterium* itself is a plant pathogen and can interfere with plant defense response to stress, resulting in misleading interpretation of data. However, by using the appropriate controls, this technique can be a powerful tool for discovering function of genes involved in strawberry–pathogen interaction, and to unravel the complex network of defense signaling pathways in this important crop.

## Concluding Remarks

Despite the large amount of molecular information originated from the use of new high-throughput technologies, providing useful data to improve strawberry crop, the knowledge of the role of most genes involved in important physiological processes is still scarce.

The advances in the last two decades of high efficiency stable and transient transformation protocols for strawberry, open new frontiers to understand the role of genes regulating important biological processes and potentially exploitable for strawberry improvement through breeding or genetic engineering.

However, the high cost, also in terms of time, of developing transgenic over-expressing or silenced lines for determining gene function, limits their usefulness to the analysis of few candidate genes. The transient transformation approach represents a rapid alternative and a highly efficient method for gene function discovery, opening new scenarios at the service of genetic engineering for strawberry crop improvement. Future research focused on the development of valuable molecular tools capable to extent the effect of transient expression, and to overcome post-transcriptional gene silencing, such as, for example, the above mentioned P19 viral protein, would be very important to allow technical advances and promote wider applications of this investigation method.

## Conflict of Interest Statement

The authors declare that the research was conducted in the absence of any commercial or financial relationships that could be construed as a potential conflict of interest.
